# Association of MC1R variation and plumage color diversity of Nigerian domestic pigeon (*Columba livia domestica*)

**DOI:** 10.5455/javar.2022.i604

**Published:** 2022-09-29

**Authors:** Xiang-Xiang Jiang, Adeniyi Charles Adeola, Foluke Eunice Sola-Ojo, Ibraheem Atolagbe Abubakar, Isiaka Hannah Fatima, Ojuerayetan Judah Olaoluwa, Abdulwahab Barakat Abodurin, Olawale Abdulafeez Olasunkanmi, Oladejo Hafsat Abisola, Oladipo Uthman, Adeshina Esther Kehinde, Hussein Hamidat, Taiwo Eyitayo Nishola, Semiu Folaniyi Bello, Min-Sheng Peng, Ya-Ping Zhang

**Affiliations:** 1 College of Life Sciences, Anhui Normal University, Wuhu, China; 2 State Key Laboratory of Genetic Resources and Evolution Yunnan Laboratory of Molecular Biology of Domestic Animals, Kunming Institute of Zoology, Chinese Academy of Sciences, Kunming, China; 3 Department of Animal Production, Faculty of Agriculture, University of Ilorin, Ilorin, Nigeria; 4 Department of Animal Genetics, Breeding and Reproduction, College of Animal Science, South China Agricultural University, Guangzhou, China

**Keywords:** *MC1R*, Missense mutations, Nigerian domestic pigeon, plumage, single nucleotide polymorphism

## Abstract

**Objectives:**

Domestic pigeons (*Columba livia domestica*) have diverse plumage pigmentations. Melanocortin 1 receptor (*MC1R*) gene variation has been correlated with color traits. The association between *MC1R* and plumage coloration in African domestic pigeons is yet to be investigated.

**Materials and Methods:**

Herein, we report the relationships between single nucleotide polymorphisms (SNPs) in *MC1R* and plumage of 35 domestic pigeons from Nigeria with 4 different plumage phenotypes plus 37 published *MC1R* sequences from France (*n =* 14) and Russia (*n =* 11).

**Results:**

We obtained 14 SNP sites among 72 individuals. Missense mutations C206T (Ser69Leu) and G253A (Val85Met) were observed in 16 and 8 Nigerian pigeons, respectively. The chi-squared test (*p <* 0.05) for C206T, G253A, and A520G has the advantage of homozygous genotypes CC, GG, and AA, respectively. The association of C206T loci showed the advantage of CC genotype in ash-red, spread, and white pigeons, and TT in blue-bar, spread, and white feather pigeons. For G253A and A520G loci, GG and AA were dominant in all plumages except for genotype AA in G253A, which was prominent in ash-red, spread, and white plumages. The three SNPs were assigned to seven haplotypes. The median-joining network revealed 20 haplotypes, including 5 in Nigeria and 2 shared.

**Conclusion:**

This study provides an insight into the association of *MC1R* variation and plumage diversity in Nigerian domestic pigeons. However, due to the limitation of the current data, we could not make further conclusions; this necessitates the need for more genomics studies on Nigerian pigeons.

## Introduction

Domestic pigeons (*Columba livia domestica*) have diverse plumage pigmentations distributed among several breeds [1]. They display extreme plumage diversity, as a result of the artificial selection of the domestic population [2]. The black plumage is due to the deposition of eumelanic pigments, while the pheomelanic pigment is responsible for the ash-red coloration in feral pigeons [3]. The variation in the sequence of the Melanocortin 1 receptor (*MC1R*) gene was found to be correlated with color traits in mammals and different poultry birds [4–14]. Despite many efforts to characterize the genetic patterns of inheritance of complex traits influencing coloration in domestic pigeons, the relationship that exists between *MC1R* and plumage coloration in African domestic pigeons (including Nigeria) is yet to be investigated. Herein, we report on our exploration of the association between single nucleotide polymorphisms (SNPs) within *MC1R* and the plumage coloration of Nigerian domestic pigeons.We identified a missense mutation C206T (Ser69Leu) related to plumage color, and this can form the basis for further molecular studies on Nigerian domestic pigeons.

## Materials and Methods

### Ethical approval

In this study, fieldwork and experiments were performed following the Guidelines of the Institutional Review Board of Kunming Institute of Zoology, Chinese Academy of Sciences (SMKY-20160105-11), and the Ethical Review Committee, University of Ilorin, Nigeria (UERC/ASN/2021/2160).

### Sample collection

We extracted genomic DNA from blood samples from 35 domestic pigeons (*C.l. domestica*) with 4 plumage color phenotypes, including ash-red (*n =* 6), blue-bar (*n =* 5), spread (*n =* 12), and white (*n =* 12), from Nigeria.

### Polymerase chain reaction and DNA sequencing

The amplification and sequencing of 866 base pair (bp) melanocortin-1 receptor (*MC1R*) were carried out (GenBank accession nos. OK318734-OK318756, OK318785-OK318769).

### Data analysis

For a comparative *MC1R* variation and diversity study on plumage color, 37 published *MC1R* sequences from France (*n =* 14) and Russia (*n =* 11) were retrieved from the GenBank [15–17]. All 72 domestic pigeon *MC1R* sequences (35 *de novo* and 37 published) were aligned and trimmed to 768 bp for analysis. The Laughing Dove (*Spilopelia senegalensis*: OK318757) was used as the out-group.

## Results and Discussion

**Figure 1.**
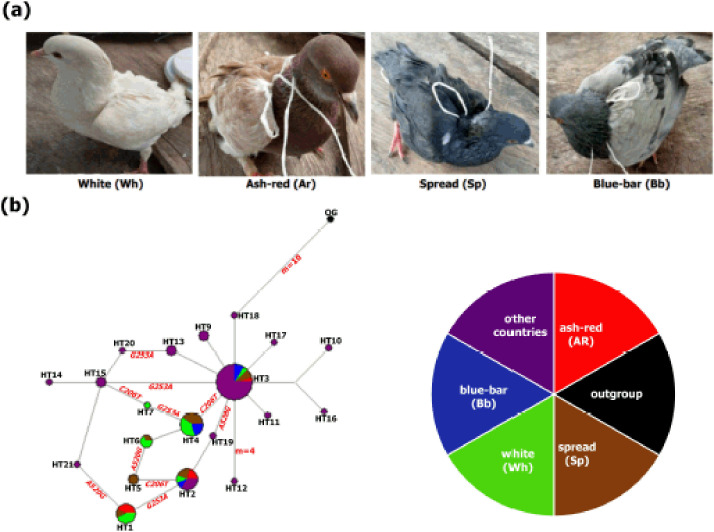
Photographs and median-joining network of *MC1R* of the four plumage categories of pigeons in Nigeria and other feral pigeons across the world. (a) Four plumage categories of the pigeons used in this study. White (Wh): the body of the pigeon is almost or completely covered with white feathers, except for a few feathers of other color; ash-red (Ar): the head and body of the pigeon are mostly covered with dark red feathers; spread (Sp): the head and body of the pigeon are completely or almost covered with black feathers; blue-bar (Bb): the pigeon’s head is covered with blue feathers, and the body parts appear mostly gray or blue feathers. (b) Median-joining network of *MC1R* of the four plumage categories of pigeons in Nigeria and other feral pigeons across the world. NETWORK v 10.2.0.0 was used [18]. The sizes of the circles are equivalent to frequencies. M in red color represents the number of mutation steps and those not indicated are just one step. Colors indicate the plumage categories. Copyright: Photographs taken by Dr. Foluke E Sola-Ojo.

In accordance with previous studies, we observed a deletion of three base pairs in all the studied *C.l. domestica*. We obtained 14 SNP sites among the 72 individuals, and 3 of them (C206T, G253A, and A520G) were more informative for Nigerian pigeons. One of the SNPs, C206T (Ser69Leu), is a missense mutation and was observed in 16 individuals of the Nigerian pigeon population. The remaining 2 SNPs, G253A and A520G, were shared with the other 37 previously published *MC1R* sequences (Table 1), particularly G253A (Val85Met) observed in 8 Nigerian pigeons has been previously reported [15]. Allele and genotype frequencies’ results showed three SNPs in the four different plumage color populations (Table 2, Fig. 1a). Based on the chi-squared test at a significant level (*p* < 0.05), three mutations of different genotype distributions in the four plumage colors revealed that C206T, G253A, and A520G have advantages of homozygous genotypes CC, GG, and AA respectively. The association of the four plumage color patterns with genotypes in C206T loci showed that CC genotype was advantageous for genotypes in ash-red, spread, and white pigeons, and TT genotype was prominent in blue-bar, spread, and white feather pigeons. For G253A and A520G loci, homozygous genotypes GG and AA were dominant in all the four plumage types except for genotype AA in G253A, which was prominent in ash-red, spread, and white plumages. The three SNPs (C206T, G253A, and A520G) were assigned to seven haplotypes (H1–H7) (Table 3). Our results revealed that haplotypes H2 and H3 occurred in all the plumage types. Similarly, haplotype H1 was observed in all plumage types except in the blue-bar plumage pigeons, while H7 was found in white pigeons. Haplotype H4 with the highest frequency was found in three plumage types except for ash-red. All the haplotypes were present in both white and brown pigeons except H7 and H5, respectively. Considering our association analysis, we observed that two haplotypes (H1 and H2) were significantly related to ash-red plumage. At the same time, H2, H3, and H4 were significantly correlated (*p* < 0.05) with spread and blue-bar plumage traits of pigeons (Table 4).

**Table 1.** 72 pigeons’ *MC1R* gene SNP sites.

Proteins SNPs	DNA SNPs	Published sequence (38)	Nigeria pigeon (35)
L42F	C124T	All samples	
S69L	C206T	0	16
V80I	G238A	All samples	
V85M	G253A	5	8
A91V	C273G	All samples	
V103M	G307A	All samples	
I122T	T365C	All samples	
V172I	G514A	All samples	
S174G	A520G	3	15
L177F	A531C	All samples	
T179I	C536T	All samples	
G192S	G574A	All samples	
C213R	T637C	All samples	
I219M	C657G	All samples	

**Table 2.** The genotype distribution of 35 Nigerian pigeons’ *MC1R* gene.

SNPs	Genotype	Ash-red	Phenotype class		White	Total	Frequency/%	*χ*^2^ value	PIC^a^
			Blue-bar	Spread					
	CC	6 (0.17)	3 (0.09)	5 (0.14)	5 (0.14)	19 (0.54)	54.29		0.3732
	CT	0 (0.00)	0 (0.00)	0 (0.00)	0 (0.00)	0 (0.00)	0.00		
SNP1 (C206T)	TT	0 (0.00)	2 (0.06)	7 (0.20)	7 (0.20)	16 (0.46)	45.71	*χ*^2^= 6.69	
	Total	6	5	12	12	35	100	*p* < 0.05	
	C	6	3	5	5	19	54.29		
	T	0	2	7	7	16	45.71		
	GG	3 (0.09)	5 (0.14)	11 (0.31)	8 (0.23)	27 (0.77)	77.14		0.2975
	AG	0 (0.00)	0 (0.00)	0 (0.00)	0 (0.00)	0 (0.00)	0.00		
SNP2 (G253A)	AA	3 (0.09)	0 (0.00)	1 (0.03)	4 (0.11)	8 (0.23)	22.86	*χ*^2^ = 6.17	
	Total	6	5	12	12	35	100	*p* < 0.05	
	G	3	5	11	8	27	77.14		
	A	3	0	1	4	8	22.86		
	GG	5 (0.14)	1 (0.03)	5 (0.14)	4 (0.11)	15 (0.43)	42.86		0.3249
	AG	0 (0.00)	0 (0.00)	0 (0.00)	0 (0.00)	0 (0.00)	0.00		
SNP3 (A520G)	AA	1 (0.03)	4 (0.11)	7 (0.20)	8 (0.23)	20 (0.57)	57.14	*χ*^2^ = 5.53	
	Total	6	5	12	12	35	100	*p* < 0.05	
	G	5	1	6	4	17	48.57		
	A	1	4	6	8	18	51.43		

PIC = Polymorphism information content

**Table 4.** Association analysis of 35 Nigerian pigeons with 7 haplotypes and with the 4 plumage types using chi-square.

Haplotype	Ash-red	Blue-bar	Spread	White	Total	*χ* ^2^
H1 (CAAG)	3 (42.86)	0	1 (14.28)	3 (42.86)	7	14.00
H2 (CGAG)	2 (33.33)	1 (16.67)	2 (33.33)	1 (16.67)	6	18.00
H3 (CGGA)	1 (16.67)	2 (33.33)	2 (33.33)	1 (16.67)	6	18.00
H4 (TGGA)	0	2 (22.22)	3 (33.33)	4 (44.44)	10	18.00
H5 (TGAG)	0	0	2 (100)	0	2	Nil
H6 (TGAA)	0	0	1 (33.33)	2 (66.67)	3	3.00
H7 (TAGA)	0	0	0	1 (100)	1	Nil
Total	6	5	12	12	35	

Numbers in bracket = percentage of each plumage color in the seven haplotypes.

**Table 3.** Distribution of 35 Nigerian pigeons’ *MC1R* gene haplotypes.

Haplotype	Number	Frequency	Ash-red	Blue-bar	Spread	White
H1 (CAAG)	7	0.200	0.086 (3)	0	0.028 (1)	0.086 (3)
H2 (CGAG)	6	0.171	0.057 (2)	0.028 (1)	0.057 (2)	0.028 (1)
H3 (CGGA)	6	0.171	0.028 (1)	0.057 (2)	0.057 (2)	0.028 (1)
H4 (TGGA)	10	0.287	0	0.057 (2)	0.115 (4)	0.115 (4)
H5 (TGAG)	2	0.057	0	0	0.057 (2)	0
H6 (TGAA)	3	0.086	0	0	0.028 (1)	0.057 (2)
H7 (TAGA)	1	0.028	0	0	0	0.028 (1)
Total	35	1				

Numbers in bracket = frequency of each plumage color in the seven haplotypes.

The median-joining network of 72 domestic pigeon *MC1R* sequences revealed 20 haplotypes, including 5 observed in Nigeria (H1, HT4, HT5, HT6, and H7), and the remaining two (HT2 and HT3) were shared with previous data (Fig. 1b).

Due to a lack of information on SNP study of *MCR1* in Nigerian poultry birds, we could not fully detail the reason for the observed significant differences between the polymorphic variants of *MC1R* and the plumage color of the Nigerian domestic pigeons sampled.

## Conclusion

This study provides initial insight into the variation in *MC1R* and plumage color diversity association of Nigerian domestic pigeons. Nevertheless, the present data in this study could not permit us to draw intensive conclusions, thereby suggesting the need for more essential genetic studies in Nigerian pigeons.

## List of abbreviations

*MC1R*, Melanocortin 1 receptor; SNP, Single nucleotide polymorphism.
